# Sensor-integrated dual-clad fiber probe for OCT-guided retinal endolaser photocoagulation

**DOI:** 10.1117/1.JBO.31.7.077001

**Published:** 2026-07-15

**Authors:** Dongyue Wu, Florian Lux, Max Mai Tobon, Holger Muenz, Soeren Schmidt, Eleonora Tagliabue, Philipp Matten, Çağlar Ataman

**Affiliations:** a University of Freiburg, Department of Microsystems Engineering, Freiburg, Germany; b Carl Zeiss AG, Corporate Research & Technology, Eggenstein-Leopoldshafen, Germany; c Carl Zeiss AG, Corporate Research & Technology, Oberkochen, Germany; d Carl Zeiss AG, Corporate Research & Technology, Jena, Germany

**Keywords:** retinal endolaser photocoagulation, optical coherence tomography, micro-optics, dual-clad fiber, surgical laser, vitreoretinal surgery

## Abstract

**Significance:**

Retinal endolaser photocoagulation is a widely performed vitreoretinal procedure but currently lacks real-time intraoperative feedback on treatment outcomes. Integrating optical coherence tomography (OCT) directly into the endolaser probe enables co-localized, intraoperative sensing of tissue response during laser delivery, offering the potential to significantly enhance surgical safety and efficacy.

**Aim:**

The aim of the study is to design, fabricate, and demonstrate a fiber-optic smart instrument with a 3D nano-printed microlens and instrument-integrated OCT sensing that enables co-localized, concurrent surgical laser delivery and tomographic sensing of tissue alterations during REPC.

**Approach:**

A dual-clad fiber was used to co-axially deliver surgical and OCT beams through the outer multi-mode and inner single-mode cores. A 3D nano-printed aspherical microlens (300  μm diameter) at the fiber tip optimized beam shaping for both lasers in the intravitreal cavity. The optical performance of the 23G instrument-integrated OCT (iiOCT) probe was characterized, and *ex vivo* validation experiments were conducted on porcine eyes.

**Results:**

Optical performance assessments demonstrated close alignment between measured and simulated beam profiles, with an OCT spot size of 37.9  μm at best focus and a surgical laser beam divergence half-angle of 6.0 deg. The probe demonstrated stable power transmission with a mean deviation of 0.35% across repeated pulses. *Ex vivo* experiments confirmed co-localized, concurrent surgical laser delivery and OCT sensing of retinal tissue alterations.

**Conclusions:**

The miniaturized iiOCT endolaser probe combines surgical laser delivery with OCT sensing, achieving adequate performance for the targeted clinical application. This sensor-integrated instrument has the potential to enable intraocular, intraoperative, and quantitative assessment of treatment outcomes. It could support feedback-driven, robotic endolaser photocoagulation, enhancing the safety and efficacy of retinal endolaser surgery.

## 1 Introduction

Retinal endolaser photocoagulation (REPC) is a well-established treatment for various retinal diseases, including proliferative diabetic retinopathy, retinal breaks, and retinal detachment.^1^^–^^5^ During the procedure, a miniaturized fiber-optic endolaser probe is introduced into the eye through a trocar to deliver laser treatment close to the retina. In current practice, treatment monitoring relies largely on microscopic observation of the lesion’s shape and size. This approach offers limited insight into the therapeutic effect and does not allow for timely dosage control during laser irradiation.^6^^,^^7^ This leads to both inadequate and excessive treatment, which is associated with complications such as retinal re-detachment and vitreous hemorrhage,^8^^,^^9^ highlighting the need for intraoperative sensory feedback in REPC procedures.

Optical coherence tomography (OCT) has been widely utilized by surgeons and clinical researchers to study tissue dynamics in slit-lamp-based REPC procedures.^6^^,^^10^^–^^13^ However, slit-lamp or microscope-integrated OCT systems positioned outside the eye have inherent challenges in aligning the OCT sensing beam with the surgical laser beam from the endolaser probe, as these systems no longer share the same optical path.^3^ Consequently, accurately registering intraoperative OCT imaging to the treatment site becomes difficult, particularly with the constantly moving endolaser probe. In addition, the OCT beam is often obstructed by the probe during tool maneuver, further limiting the feasibility of such approaches. In contrast, an instrument-integrated optical coherence tomography (iiOCT) sensor that enables intraocular sensing and surgical laser delivery within the same instrument is ideal for intraoperative sensory feedback.^14^^–^^17^ Yet the limited space and the need to couple light across different wavelengths, NAs, and power levels make the opto-mechanical design of such probes challenging. Operation in the intravitreal cavity requires extreme lens geometries to achieve sufficient refractive power, making conventional lensed fibers less suitable. Similarly, a single GRIN lens^18^ is suboptimal for simultaneously focusing the OCT beam at the desired working distance while confining the rapidly diverging surgical laser beam within the lens aperture under the size constraints imposed by this clinical application (see Supplementary Material 1).

Three-dimensional (3D) nano-printing via two-photon polymerization offers significant design flexibility and precision, enabling rapid prototyping of advanced micro-optical elements and systems.^19^^–^^22^ This technology has unlocked new potential for sensor design in the fields of biomedical imaging and microsurgery, including applications such as intravascular imaging,^23^^,^^24^ brain tumor detection,^25^ and retinal surgery.^26^ 3D nano-printed opto-mechanical components can be easily integrated into compact surgical instruments while offering high imaging quality and various sensing modalities.

In this work, we present an iiOCT probe that combines endolaser delivery coaxially within the same instrument via a dual-clad fiber (DCF), using a 3D nano-printed microlens for beam shaping and a free-space fiber coupling setup to facilitate power transmission from two light sources. As a proof of concept, we investigate the ability of the iiOCT sensor to detect retinal tissue morphological changes in REPC. The probe’s beam profiles, optical power transmission, and long-term stability under high-power surgical laser pulses were characterized. Its capability for co-localized OCT sensing and surgical laser delivery was demonstrated through *ex vivo* experiments in porcine eyes. Together, these results establish a foundation for future intraocular, intraoperative feedback systems that could enable quantitative assessment and feedback-controlled retinal endolaser surgery. Sections of this work, specifically earlier increments, have been previously published in Optica Open.^27^

## 2 Methods

### 2.1 Combined Sensing System

**Fig. 1:**
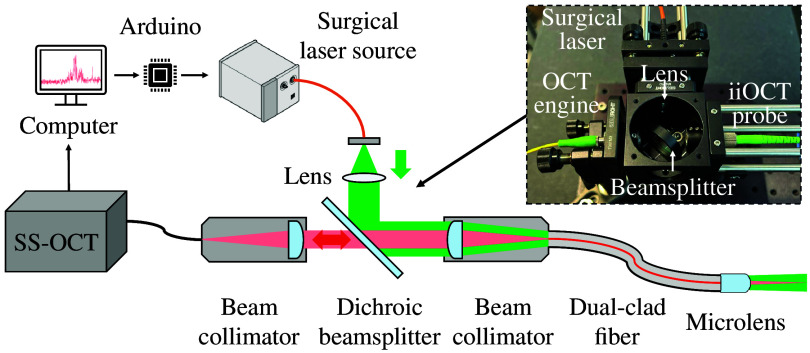
Schematic of the combined iiOCT endolaser probe and free-space fiber coupling setup.

The endolaser probe consists of a DCF (F-SMM900/007, Fibercore, Southampton, United Kingdom) and a 3D nano-printed microlens at the distal end. The DCF has a single-mode core with a mode-field diameter of ∼6  μm at λ=1060  nm and a multi-mode first cladding with 100  μm diameter, allowing both the single-mode OCT detection beam and the multi-mode surgical laser beam to be guided through one fiber. To couple the light from a swept-source OCT (SS-OCT) engine (SL10 MEMS-VCSEL Swept Source, 1060 nm, 100 kHz, Thorlabs GmbH, Bergkirchen, Germany) and a 532 nm surgical laser source (ZEISS VISULAS^®^ green, Carl Zeiss Meditec AG, Jena, Germany) into the DCF probe, a free-space fiber coupling setup was designed. A pair of fixed-focus fiber collimators (F220APC-1064, Thorlabs GmbH, Bergkirchen, Germany) were connected to the sample arm of the SS-OCT engine and the endolaser probe, respectively, to couple the OCT beam into the probe. An f=10  mm lens (LA1116-A, Thorlabs GmbH, Bergkirchen, Germany) was used to collimate the surgical laser beam delivered via a multi-mode fiber with a 50  μm core diameter (M14L02, Thorlabs GmbH, Bergkirchen, Germany) and to compensate for the defocus caused by the collimator. The two optical paths were combined using a longpass dichroic mirror (DMLP650, Thorlabs GmbH, Bergkirchen, Germany) placed at a 45 deg angle in the OCT path. The alignment tolerance analysis of the free-space fiber coupling setup is presented in Supplementary Material 2. An Arduino Mega 2560 microcontroller was used to bridge the connection between the surgical laser source and the computer to enable computer-controlled triggering of the laser delivery signal via script. Figure 1 shows the schematic of the setup.

### 2.2 Optomechanical Design of the Sensor Head

A 3D nano-printed micro-optical element was designed and fabricated for guiding both the OCT and surgical laser beam onto the retina. The design goals were to achieve a focused OCT beam with a working distance of 2 to 3 mm in the intravitreal cavity and to produce a surgical laser beam with a divergence smaller than that of conventional endolaser probes (typically ≥9  deg half-angle). Several boundary conditions were considered when defining the lens geometry. First, the diameter of the lens should not exceed the inner diameter of the straight tubing used for the 23G surgical instrument (0.33 mm inner diameter). Second, the diameter of the surgical laser beam expanded faster than that of the OCT beam inside the lens, as the first cladding had both a higher NA (0.22) and a larger diameter than the core. Consequently, the surgical laser beam could easily exceed the diameter of the lens and cause power loss from the side walls. To meet these requirements without increasing manufacturing complexity from multiple lens surfaces, a plano-convex lens was designed and optimized with commercial ray-tracing software (ZEMAX OpticStudio) in sequential mode. The optimization was performed at both 1060 nm (primary) and 532 nm wavelengths, a photoresist refractive index of 1.511^28^ and a propagation medium refractive index of 1.33 (water).

**Fig. 2:**
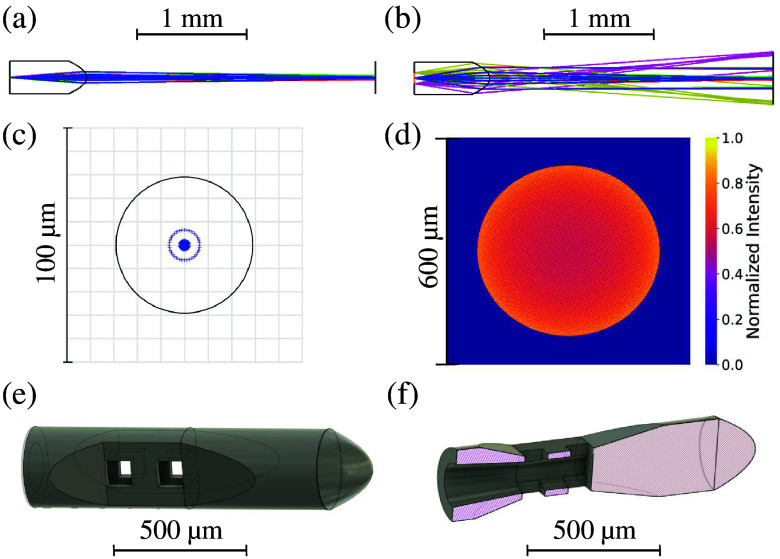
(a) and (b) Zemax simulation of OCT beam (a) and surgical laser beam (b) propagation. (c) and (d) Simulated spot diagram of 1060 nm beam (c) with Airy Disk (black) and 532 nm beam (d) at 2.67 mm from lens surface. (e) and (f) CAD model with section view of the 3D printed sensor head.

Figures 2(a) and 2(b) show the ZEMAX simulation of the lens. The lens has a diameter of 300  μm and a thickness of 710  μm. This lens thickness was selected to balance two competing requirements. First, it provides sufficient propagation length for the OCT beam to achieve the target image-space NA at the lens exit surface. Second, it keeps the more rapidly diverging surgical laser beam within the lens aperture, avoiding power loss at the side walls. The convex surface is aspherical with a radius R=−0.066  mm and a conic constant κ=−1.021. For the OCT path, the image space NA is 0.03 and the working distance is 2.67 mm, with a depth of focus of ∼1.6  mm. For the surgical laser path, the divergence of the beam is 5.8 deg half-angle in simulation. The surgical laser spot size on the retina is determined by the beam divergence, the tool-to-retina distance, and the initial beam size at the probe tip. A small divergence angle improves the stability of beam size and energy distribution across variations in tool-to-retina distance.^29^ Moreover, the ability of iiOCT to provide precise distance measurements could enable monitoring and control of the tool-to-retina distance,^30^ allowing more flexible beam-size adjustment and improving the repeatability of laser spot size and energy distribution across pulses.

The CAD model of the complete element is shown in Figs. 2(e) and 2(f). A 600  μm long hollow channel with an insertion funnel was extended from the plane surface of the lens for passive alignment with the DCF. This design includes four side wall openings to facilitate the removal of uncured photoresist after 3D nano-printing. These openings also improve visualization of the fiber tip during assembly.

### 2.3 Lens Fabrication and Probe Assembly

**Fig. 3:**
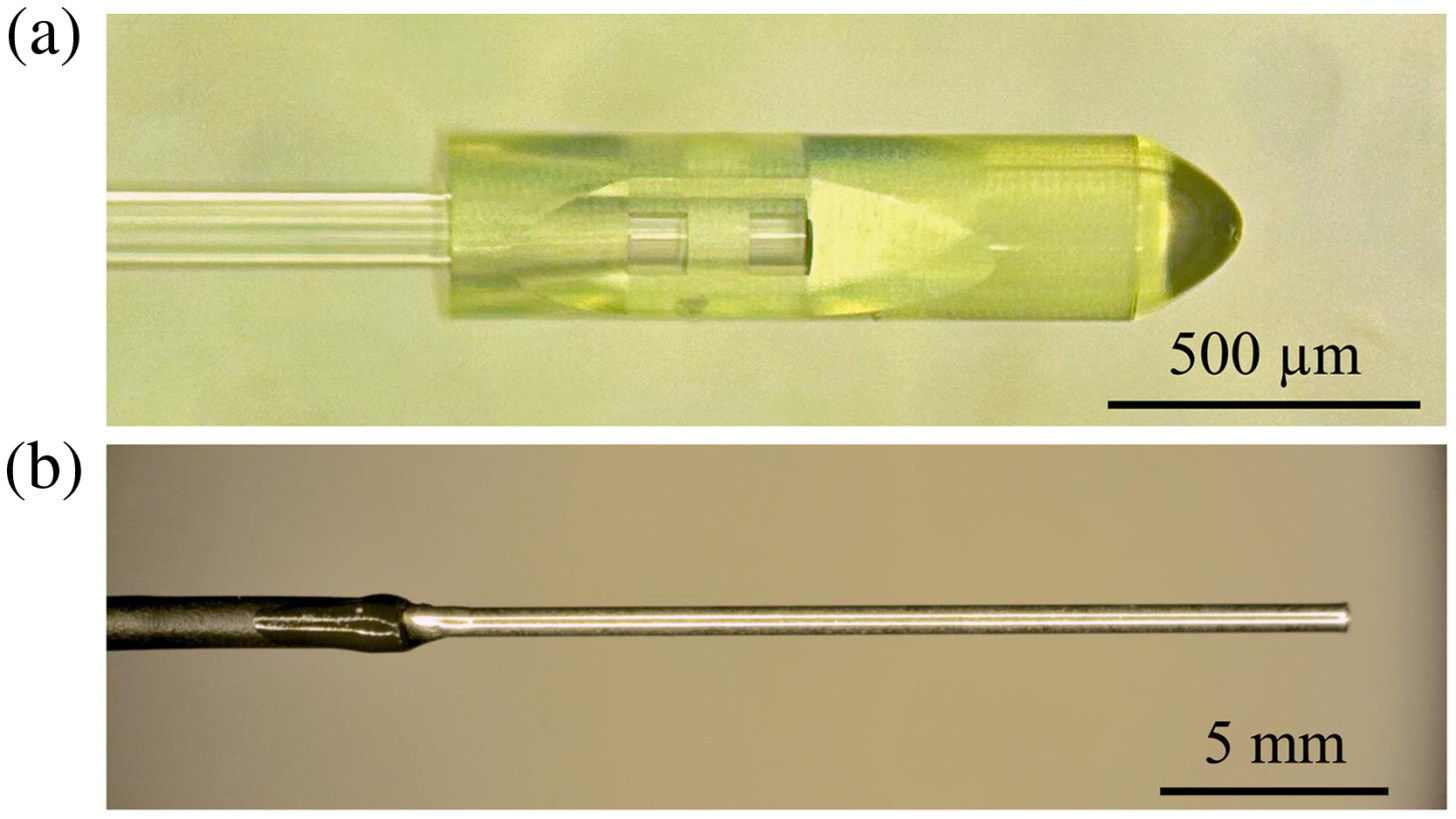
(a) Microscopic image of the probe tip. (b) Probe assembly in 23 G metal tube.

The microlens was printed with IP-S photoresist (Nanoscribe GmbH, Eggenstein-Leopoldshafen, Germany) onto ITO-coated fused silica substrates using a commercial 3D nano-printer (Photonic Professional GT+, Nanoscribe GmbH, Eggenstein-Leopoldshafen, Germany) with a 25× objective (25×/0.8 Imm Corr DIC M27, Carl Zeiss AG, Oberkochen, Germany) in a dip-in configuration. The laser power was adjusted to 25 mW at a scanning speed of 50  mm/s. For the convex surface of the lens, the slicing distance was set to 0.1  μm and the hatching distance to 0.5  μm. For the insertion channel and cylindrical part of the lens, the slicing distance was set to 1  μm and the hatching distance to 0.5  μm to reduce overall print time. The printed microlens was then developed and UV-cured and was manually assembled onto a 1 m long DCF with a cleaved tip, secured in place using UV-curing optical adhesive (Loctite AA 3301, Henkel AG, Düsseldorf, Germany). The probe tip was then protected by a rigid 23 G metal tube. The printed microlens and the fiber probe assembly are presented in Fig. 3.

## 3 Results

### 3.1 Lens Characterization

**Fig. 4:**
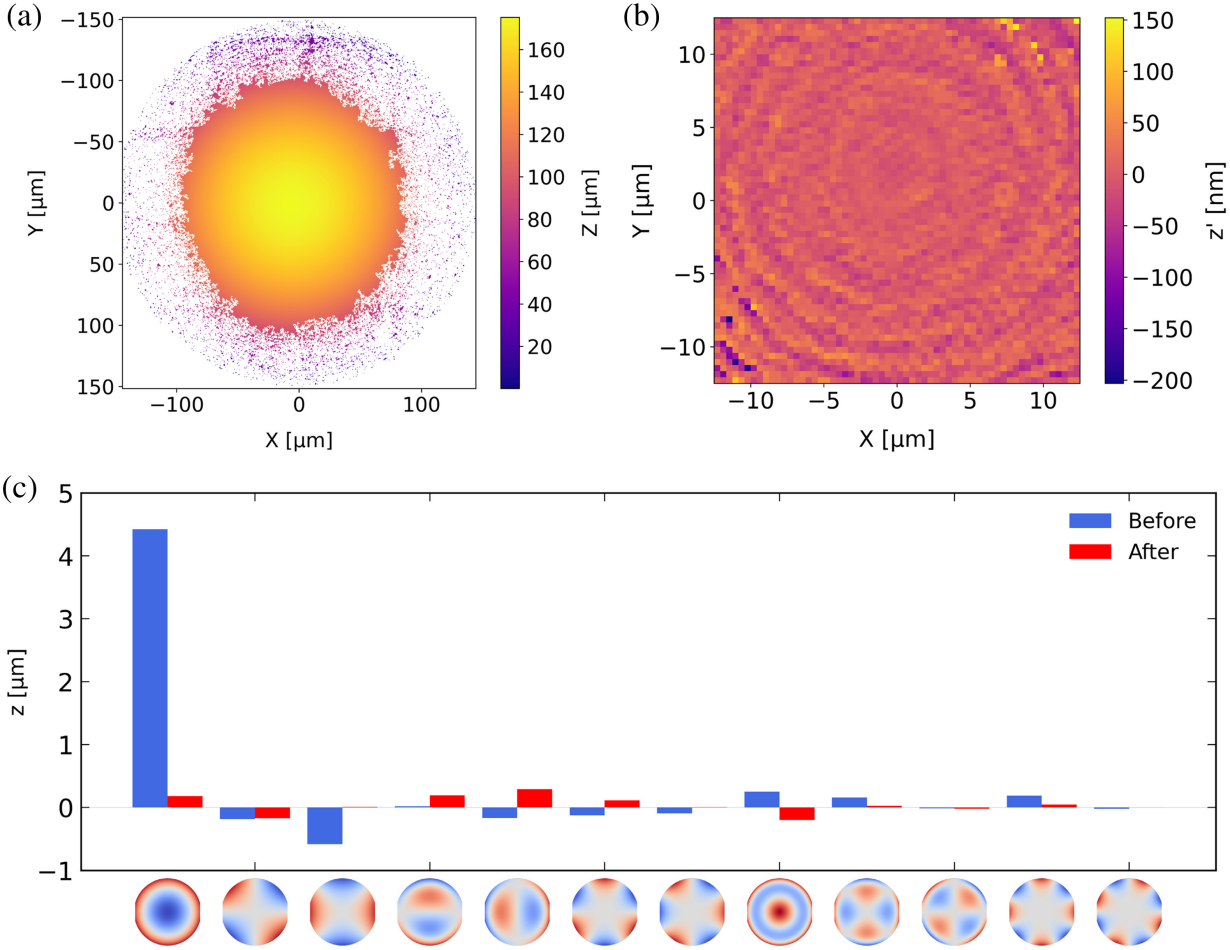
(a) Measured surface profile of the convex surface. (b) High-pass filtered section of the lens ($R_{\mathrm{RMS}}=21.47\;\mathrm{nm}$). (c) Zernike polynomials differences between measured and designed surface before and after shape compensation.

The surface shape and roughness of the printed elements were characterized using a 3D optical profiler (NewView™ 9000, Zygo Corporation, Middlefield, Connecticut, United States) operated in aperture-stitching mode, with the resulting surface topology shown in Fig. 4(a). For the convex surface of the lens, the root-mean-square roughness ($R_{\mathrm{RMS}}$) was measured within a central square region of $25\times 25\;\mu m^{2}$ [Fig. 4(b)], where sufficient interferometric data coverage was available. Within this area, a high-pass filter with a cut-off period of 25  μm (*EN ISO 25178:2012*) was applied. The results indicate an $R_{\mathrm{RMS}}$ of 21.47 nm.

During lens fabrication, structural shrinkage occurring during polymerization and subsequent development processes can introduce shape deviations in the optical components.^31^ To quantify these deviations, standard Zernike polynomials were fitted to both the measured and ideal surface profiles, and the difference between the two was calculated. To compensate for the shape deviation, the fitted deviation terms were incorporated into the original optical design, and the revised design was reprinted. The results [Fig. 4(c)] demonstrate the improvement in shape accuracy after five iterations, e.g., the difference in $Z_{2}^{0}$ decreased from 4.424 to 0.183  μm, and $Z_{2}^{2}$ was reduced from 0.585 to 0.008  μm.

**Fig. 5:**
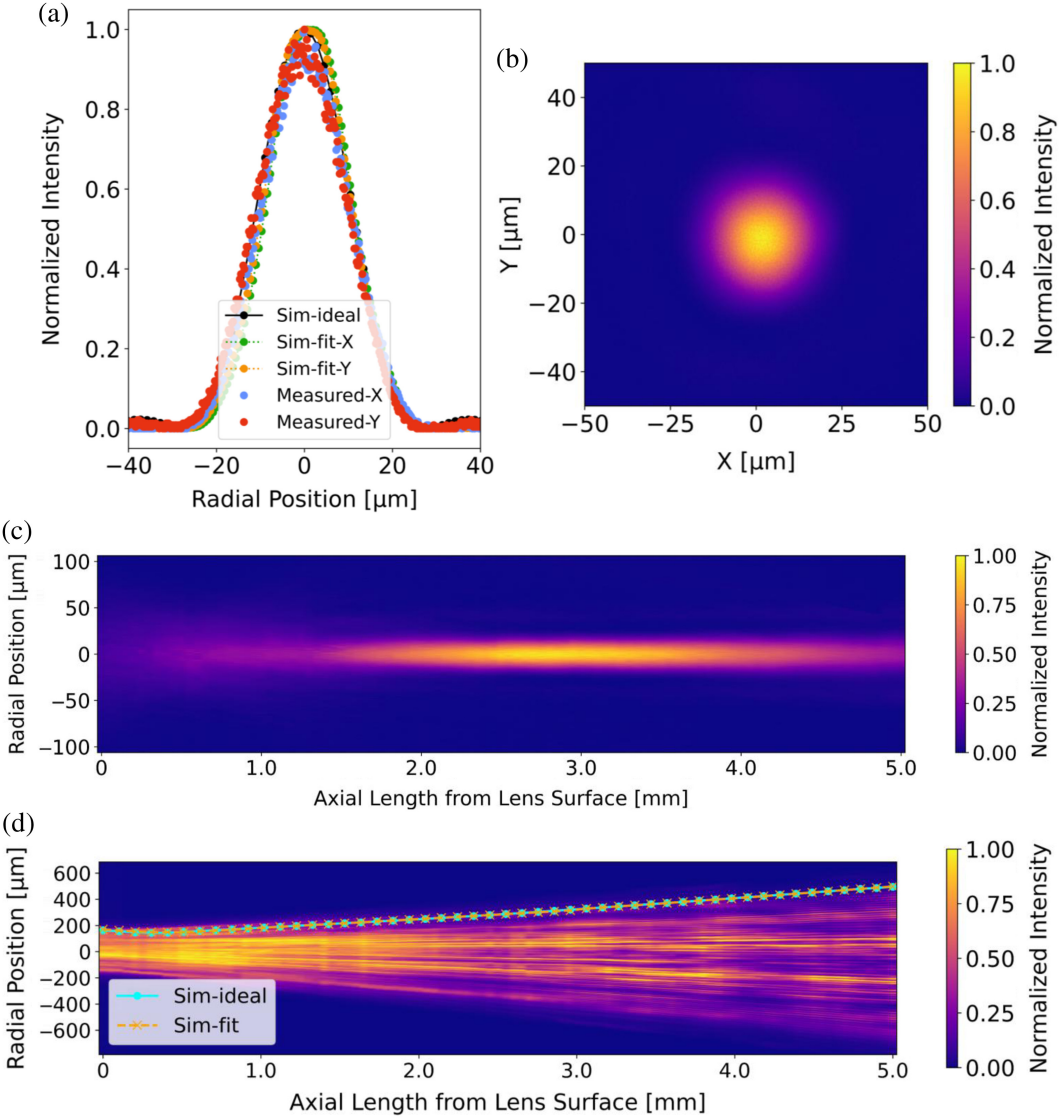
(a) Cross-sectional intensity profiles of the 1060 nm beam at best focus from design simulation, Zernike polynomials fit simulation, and measured beam. (b) Focal spot of the 1060 nm beam. (c) and (d) Beam profile along the optical axis of the (c) 1060 nm and (d) 532 nm path.

To evaluate the optical performance of the lens, the probe was connected to a 1060 nm swept source (Axsun, Excelitas Technologies Corp., Pittsburgh, Pennsylvania, United States) and the beam was imaged in water using a 25× objective lens (25×/0.8 Imm Corr DIC M27, Carl Zeiss AG, Oberkochen, Germany) in conjunction with an infinity-corrected tube lens (f=160  mm, LINOS GmbH, Mainz, Germany) and captured with a CCD camera (UI-1240SE-NIR, IDS GmbH, Obersulm, Germany). The beam profiles are shown in Fig. 5. The $1/e^{2}$ spot size of the beam at best focus was 37.9  μm in the x-direction and 37.49  μm in y-direction, which are close to simulation results of the design (37.7  μm) and the optical surface defined by Zernike polynomials from surface profile measurement (36.3  μm in x and 36.7  μm in y). The beam profile was also recorded from 0 to 5 mm from the lens surface with 50  μm step size, and is shown in Fig. 5(c). The best focus was found at 2.60 mm, which is close to simulation from design (2.67 mm) and the Zernike polynomial fit surface (2.64 mm). The beam profile of the 532 nm path, obtained using a 532 nm laser source (CPS532-C2, Thorlabs GmbH, Bergkirchen, Germany) and a CMOS camera (U3-3882LE Rev.1.2, IDS GmbH, Obersulm, Germany), is shown in Fig. 5(d) with simulated beam radius from the ideal and Zernike polynomial-fit surface. The calculated beam divergence half-angle was ∼6.0  deg, which is virtually identical to the simulation results (5.8 deg). The differences between the ideal and measured beam shape, size and working distance are attributed to manufacturing errors of the lens [Fig. 4(c)], variations in the NA of the DCF, and assembly tolerances.

### 3.2 Optical Power Transmission in OCT and Surgical Laser Paths

The coupling efficiency of the optical power for both paths was examined by measuring the optical power at the probe tip using a photodiode power sensor (S121C, Thorlabs GmbH, Bergkirchen, Germany) and comparing it with the input power of the respective sources. The input power of the OCT path was measured from the sample arm of the SS-OCT engine before being connected to the free-space setup. For the surgical laser, the input power was verified by direct measurement at the tip of the input multimode fiber prior to connection to the free-space setup. The measured values were in close agreement with the control panel settings of the laser source, which were therefore adopted as the input power reference. On average, the optical power transmission of the surgical laser path was 50.95%. With a maximum output power of 1500 mW from the surgical laser source, this efficiency is sufficient to cover the power range typically required for REPC.^32^ The OCT path achieved an average coupling efficiency of 77.86% in a single direction (from the OCT engine to the probe tip).

**Fig. 6:**
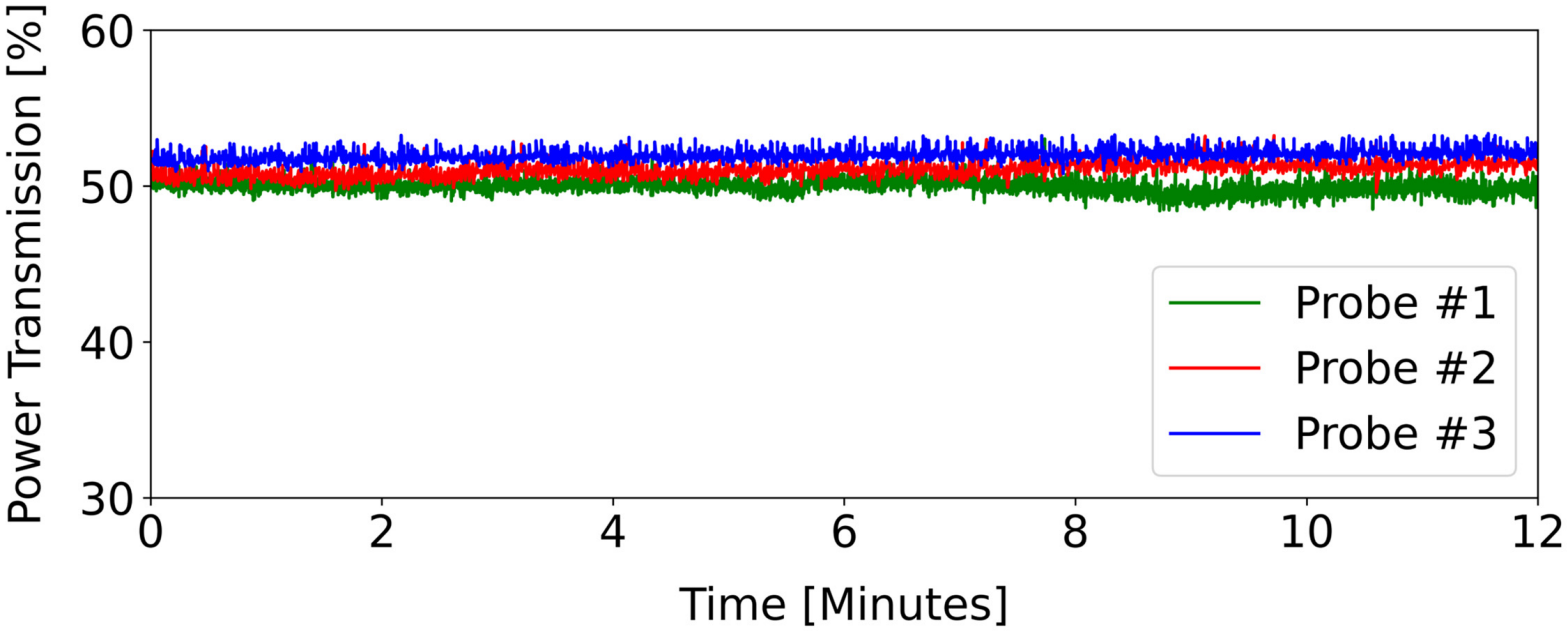
Optical power transmission (λ=532  nm) of the probes in repetitive pulses.

To investigate the long-term stability of the probe when repetitive pulses are delivered at short intervals in surgical scenarios, we measured the optical power at 532 nm from the probe tip with an input pulse power of 800 mW from the surgical laser source, a pulse duration of 200 ms and a 500 ms interval between pulses, which resembles the pulse rate used in clinical applications.^32^^–^^34^ Each test lasted for 12 min with ∼1028 pulses, and the power transmission of all probes is presented in Fig. 6. It was observed that all three probes demonstrated stable transmission, with the mean absolute deviation being 0.35%. Similar stability was observed when measuring the OCT transmission before and after the stability test, with average transmission recorded at e.g., 77.64% before the test and 77.86% after the test.

### 3.3 OCT System Characterization

**Fig. 7:**
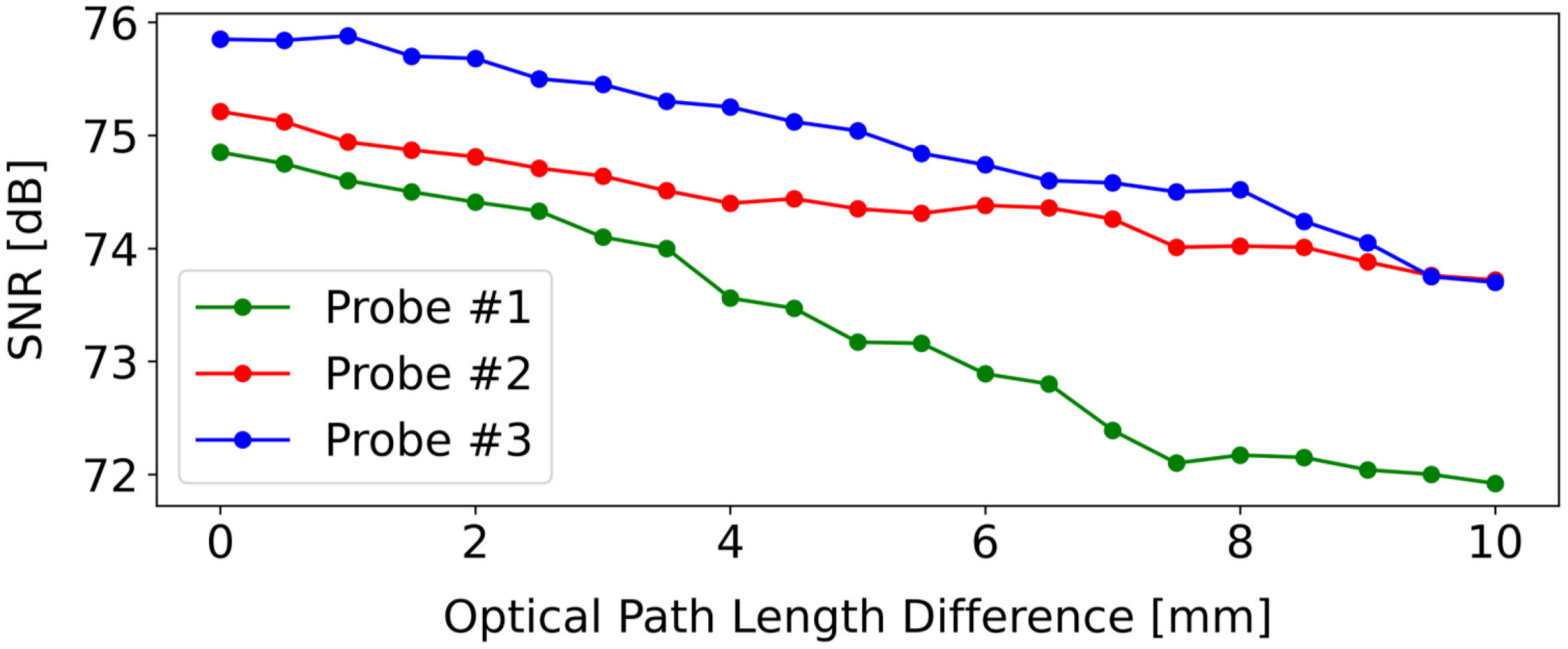
SNR measurements of the optical system.

The iiOCT probe and the free-space coupling system are additional components attached to the sample arm of the SS-OCT system. These optical components collectively influence the modulation transfer function, which can potentially limit the OCT signal strength at higher spatial frequencies and thereby the sensitivity. To assess the sensitivity for high spatial frequency features, we measured the system signal-to-noise ratio (SNR) roll-off using a mirror sample immersed in water, which generates an SNR equal to the sensitivity of the OCT system.^35^^,^^36^ The mirror was placed in the focal plane of the probe and remained stationary during the measurement. The sample arm power was attenuated to 1.62 mW (measured at the probe tip), and the reference arm power was adjusted accordingly to obtain the highest achievable SNR. The OCT peak of the mirror was moved using a dynamic delay line in the reference arm from 0 to 10 mm optical path length in the image frame. The results are shown in Fig. 7(a), indicating an average of 2.2 dB decrease from the respective highest SNR value of each probe within the range of 0 to 10 mm.

### 3.4 *Ex Vivo* Validation

**Fig. 8:**
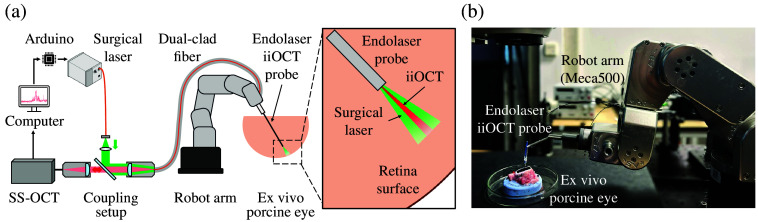
*Ex vivo* experiment setup: (a) schematic illustrating the co-localized surgical laser and iiOCT sensing beam, and (b) corresponding photo of the experimental setup.

To evaluate the combined surgical laser delivery and iiOCT sensing capability in REPC, we conducted *ex vivo* validation on open-sky porcine retina. The porcine eyes were obtained as by-products from a local abattoir and were used within 24 h of enucleation. All experiments were performed on *ex vivo* samples and therefore did not require additional institutional ethics approval. The anterior segment of the eye was removed shortly before the experiment to gain easier access to the retinal surface. Regions free of major vessels, optic nerve head, and visible retinal tear of detachment were selected to minimize confounding effects. The endolaser probe was steered by a 6-degrees of freedom robot arm (Meca500, Mecademics, Montreal, Canada) with 5  μm motion precision. The robot was controlled via a custom Python script, which commanded predefined lateral movements between treatment sites and temporarily held the probe stationary during each iiOCT M-scan acquisition. The output power of the OCT path at the iiOCT probe tip was measured to be 4.02 mW, therefore falling below the ANSI Z136.1^37^ maximum permissible exposure of 4.31 mW (see Supplementary Material 3). The present work demonstrates the feasibility of the combined sensing concept and is not intended as a clinical-grade device. The experimental setup is shown in Fig. 8.

Prior to laser delivery, the endolaser probe was driven close to the retinal surface by the robot arm. The distance between the probe-tip signal peak and the retinal surface along the tool axis was adjusted using real-time iiOCT A-scans to align the retina with the iiOCT focal plane (∼2.6  mm) for optimal sensing. Once the distance was adjusted, the probe was temporarily fixed in place by the robot arm, and a trigger signal was sent to the OCT engine. Consecutive iiOCT A-scans (i.e., an iiOCT M-scan) were acquired using 1800 A-scans at a sampling interval of 0.8 ms, yielding a total temporal window of 1440 ms per acquisition. The surgical laser was triggered ∼250  ms after the iiOCT acquisition started, ensuring full capture of the retinal structure pre-, during-, and post-laser delivery. After OCT M-scan acquisition, the probe was moved by the robot arm laterally to the next site by 1 mm to avoid energy overlapping,^38^ and the experimental protocol was repeated. Four laser spots were placed in each *ex vivo* sample, corresponding to probe-tip power levels of 100, 200, 300, and 400 mW. To account for biological variations in retinal thickness, pigmentation, and other tissue properties, this entire procedure was repeated across seven different eyes.

**Fig. 9:**
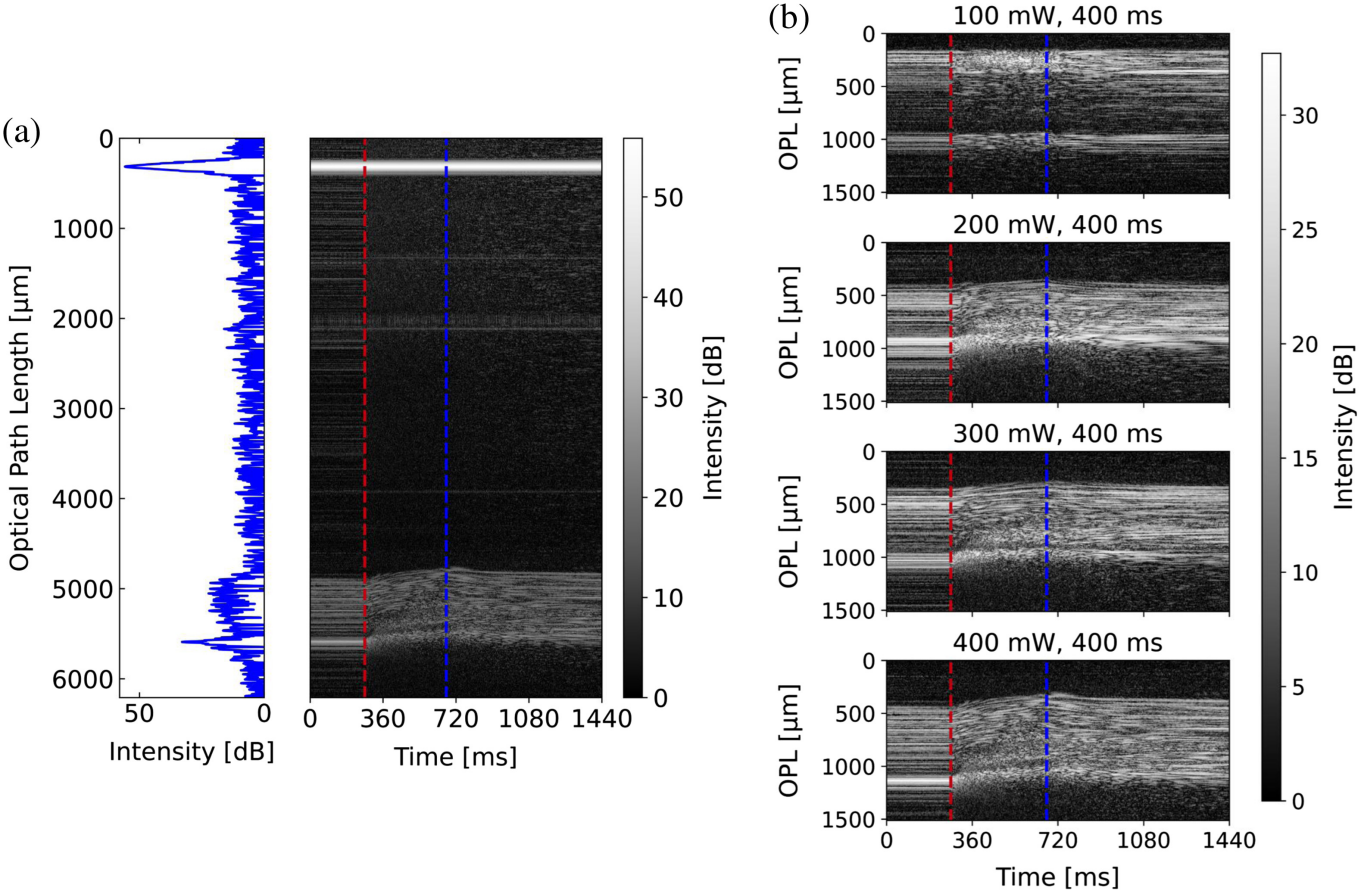
(a) An iiOCT M-scan of a porcine retina during surgical laser delivery. The red and blue vertical lines indicate the start and end of laser delivery, respectively. (b) M-scans of four laser-irradiated sites with different applied laser powers, showing different levels of tissue change observable in the structural OCT signal. The power values were determined by multiplying the input power by the system’s transmission (Video S1, MP4, 8.84 MB [URI: https://doi.org/10.1117/1.JBO.31.7.077001.s1]; Video S2, MP4, 8.86 MB [URI: https://doi.org/10.1117/1.JBO.31.7.077001.s2]).

Figure 9(a) shows an iiOCT M-scan at a single laser delivery site. The laser power was set to 800 mW at the source, which corresponds to ∼400  mW at the probe tip, for a pulse duration of 400 ms. A large region of interest encompassing both the probe tip and the retina was displayed, where interfaces between the DCF and microlens (at ∼400  μm), the lens and vitreous (at ∼1400  μm), and the porcine retina were clearly visible. Artifacts and ghost images were also observed (e.g., 2000 to 4000  μm). Clear boundaries between the vitreous and retinal surface (at ∼4800  μm), along with a distinguishable retinal pigment epithelium (RPE) layer indicated by a strong signal peak within the retina (at ∼5600  μm), could be observed. Tissue changes, including layer thickening and surface displacement, indicated that the iiOCT probe delivered sufficient energy to induce alterations and possessed adequate sensitivity to detect tissue responses in REPC. The onset of tissue change was marked by a distinct alteration in the structural OCT signal. During laser delivery, the retina lost its layered appearance. The hyporeflective band above the RPE became perturbed, and the retinal surface displayed noticeable displacement (see Video S1). These observations are consistent with tissue responses reported in other slit-lamp-based photocoagulation systems,^6^^,^^11^^,^^13^ indicating that the probe performed concurrent iiOCT sensing at the exact laser-irradiated location.

To further demonstrate the probe’s sensing capability, iiOCT M-scans of retinal tissue under different laser powers are shown in Fig. 9(b). The laser power was set to 200, 400, 600, and 800 mW at the source, corresponding to ∼100, 200, 300, and 400 mW output at the probe tip, with a pulse duration of 400 ms. All laser-irradiated sites exhibited morphological deformation shortly after the onset of irradiation (see Video S2). At the lowest power (100 mW), tissue morphology remained largely stable throughout the pulse. As the laser power increased, the layered structure became progressively more perturbed. The magnitude of surface displacement also increased markedly, reflecting different levels of tissue response corresponding to the applied power. Overall, preliminary qualitative assessment of the iiOCT M-scans demonstrated a strong power dependence of tissue change, indicating that the probe is capable of capturing and revealing tissue alterations relevant to REPC.

To provide further quantitative analysis, the retinal surface depth in each iiOCT M-scan was extracted using a gradient-based segmentation algorithm. The relative surface displacement $\Delta z_{s}$ with respect to the pre-laser baseline depth was calculated for all M-scans as

$$\Delta z_{s}(t)=|z_{s}(t)-z_{s}(t_{\mathrm{pre}})|.$$ (1)

**Fig. 10:**
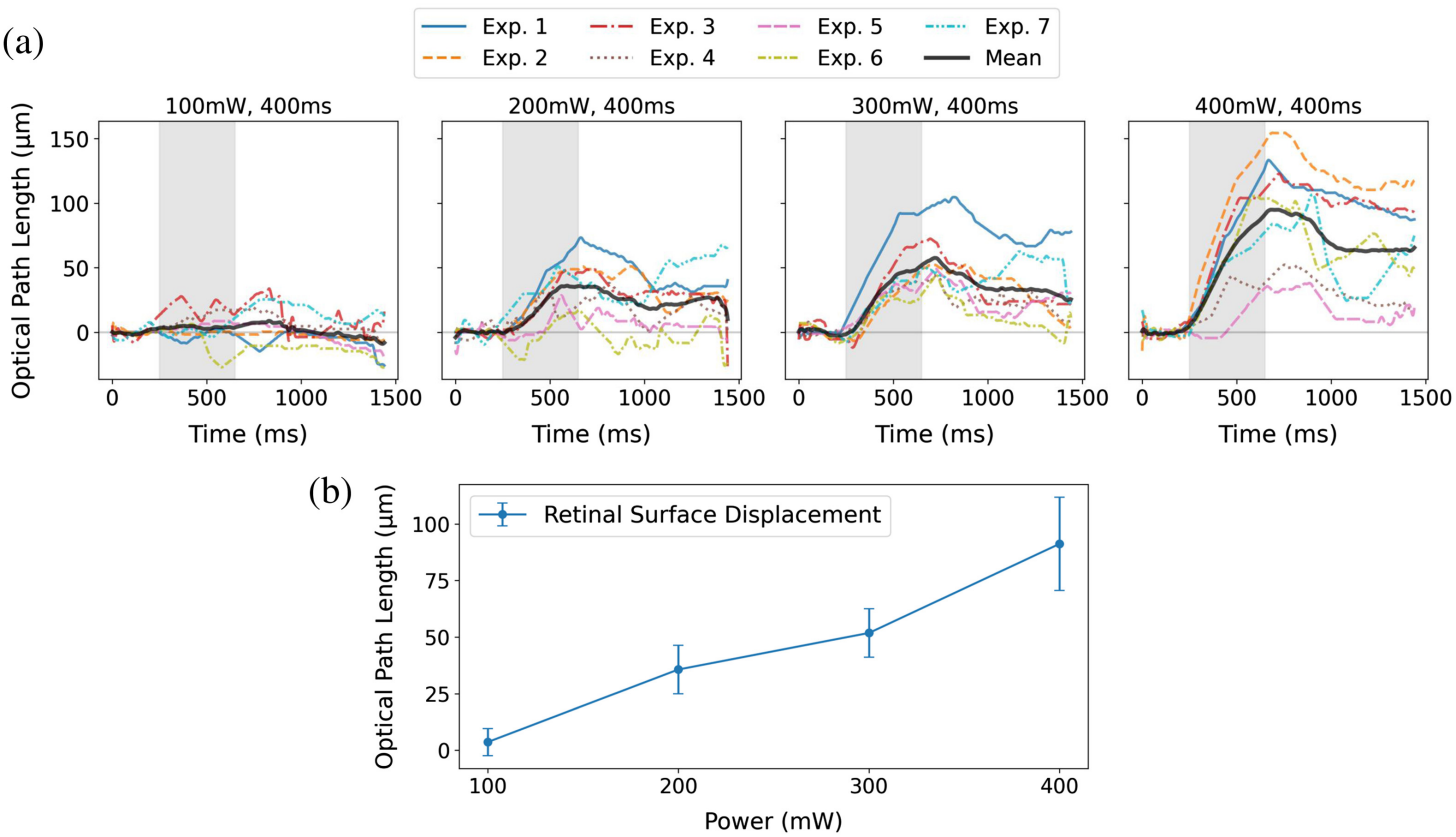
Quantitative retinal surface displacement analysis. (a) Temporal evolution of retinal surface displacement for all seven experiments at each power level, with the mean trace overlaid in black. The horizontal gray line indicates the pre-laser baseline, and the shaded region indicates the laser delivery window. (b) Mean retinal surface displacement at pulse end as a function of applied laser power. Error bars represent the standard deviation across seven experiments.

The results across all seven experiments are presented in Fig. 10. Figure 10(a) shows the temporal evolution of retinal surface displacement for each experiment and power level, with the mean trace overlaid. At the lowest power level (100 mW), surface displacement remained near zero throughout the M-scan, with traces fluctuating around the pre-laser baseline. At medium and high power levels (200 to 400 mW), a pronounced increase in surface displacement was observed following laser onset, with the magnitude scaling with applied power. The displacement reached its maximum near or shortly after the end of the pulse, followed by a partial return toward the pre-laser baseline. The retinal surface did not fully recover within the OCT acquisition window, suggesting either incomplete thermal relaxation or persistent structural change. Figure 10(b) shows the mean surface displacement at pulse end as a function of applied laser power, with error bars representing the standard deviation across the seven experiments. The results demonstrate a clear and monotonically increasing relationship between applied laser power and retinal surface displacement. This quantitative power-response characterization confirms that the iiOCT probe is capable of resolving graded tissue responses corresponding to different surgical laser powers.

## 4 Discussion

In this work, a miniaturized iiOCT endolaser probe was developed using a DCF and a 3D nano-printed microlens to enable combined OCT sensing and surgical laser delivery in REPC. The optical element demonstrated near-diffraction-limited performance and shaped both optical paths as designed. The free-space fiber coupling setup facilitates sufficient power transmission from both light sources into the probe, and the transmission is stable over repetitive pulse delivery. The probe’s concurrent OCT sensing and surgical laser delivery capabilities were demonstrated through *ex vivo* experiments, confirming its performance is adequate for the target clinical application. Nonetheless, there is potential for further improvement in both the probe design and the OCT system.

First, in our *ex vivo* experiments, the probe remained stationary while a surgical laser pulse was delivered, allowing iiOCT acquisition of the tissue response at the laser-irradiated site. No optical or robotic scanning of the iiOCT beam was performed during laser delivery. This approach enabled uniform acquisition of discrete OCT data packages at a fixed location (i.e., in the center) for each treatment area, thereby simplifying the post-processing and analysis workflow. Although the iiOCT signal during tool movement between treatment sites was not fully utilized in this study, it could be leveraged to measure and robotically control the distance between the tool and the retina during probe maneuver, similar to the process described in Sec. 3.4 prior to laser delivery. Such distance measurements and feedback could help maintain safe tool positioning and proper tool orientation between treatment areas, which could enhance procedural safety.

Second, the image artifacts in the OCT signal were mainly attributed to multimodal crosstalk within the DCF. The back-scattered light that travels in the first cladding of the DCF (with a lower effective refractive index than that of the fundamental mode) can interfere with the OCT reference arm and create ghost images of the sample.^39^^–^^41^ These artifacts could be reduced by working in a liquid environment or by using longer sections of DCF to create an optical path length delay for the artifact signal that is higher than the depth of the OCT imaging range.^42^ In addition, the signal peak originated from the DCF to microlens interface (e.g., between 1000 and 1500  μm) could be further reduced by applying anti-reflection coatings on the microlens.

Third, in the sensitivity characterization of the OCT system, the SNR exhibited an average decrease of 2.2 dB over a 10 mm optical path length. Although sensitivity roll-off at longer path lengths could pose challenges for distance sensing, system performance was adequate at the validated operating distance of ∼2.6  mm. This was sufficient to detect tissue morphological changes across the range of applied surgical laser power levels, as demonstrated by the *ex vivo* results in Sec. 3.4.

Finally, the use of open-sky *ex vivo* porcine eyes differs from *in vivo* clinical conditions in several respects relevant to clinical translation. Physiological dynamics such as respiratory and cardiac motion, blood circulation, temperature regulation, and tissue healing were absent in our *ex vivo* model. *In vivo* experiments will therefore be essential to fully characterize tissue response under realistic conditions. Integrating real-time motion correction algorithms could further help suppress motion artifacts and maintain signal stability. The use of a robotic arm with motion scaling and tremor reduction could additionally facilitate probe maneuvering in future *in vivo* experiments. Translating findings from porcine to human eyes will also require careful consideration of anatomical differences, including retinal layer thickness and pigmentation,^43^ both of which influence surgical laser energy absorption and iiOCT signal strength. Addressing these physiological and anatomical factors in future experiments will be important for ensuring robust and generalizable performance across diverse clinical settings.

## 5 Conclusion

In this work, we present a miniaturized iiOCT endolaser probe utilizing a DCF and a 3D nano-printed microlens. By integrating an OCT sensing modality directly into the surgical instrument, the probe enabled intraocular, co-localized iiOCT sensing and surgical laser delivery during REPC. Future work will focus on improving the coupling efficiency of the free-space setup, reducing the lens dimensions for smaller surgical instruments, and further processing and analyzing the iiOCT signal in biosamples. The ability to detect tissue responses intraocularly during endolaser procedures represents a significant advancement in sensor-guided vitreoretinal surgery. It could enable quantitative analysis of retinal responses, enhance understanding in areas such as dosimetry, and open possibilities for feedback-driven robotic surgery. By providing real-time iiOCT sensing during REPC, this sensor-integrated optical smart instrument has the potential to improve safety, reduce complications, and ultimately elevate treatment outcomes.

## Supplementary Material

10.1117/1.JBO.31.7.077001.s01

10.1117/1.JBO.31.7.077001.s02

10.1117/1.JBO.31.7.077001.s03

10.1117/1.JBO.31.7.077001.s1

10.1117/1.JBO.31.7.077001.s2

## Data Availability

Data underlying the results presented in this study are not publicly available but may be obtained upon reasonable request.
