# Supplementary material for: Sensor-integrated dual-clad fiber probe for OCT-guided retinal endolaser photocoagulation
Source: J Biomed Opt. 2026 Jul 15;31(7):077001. doi: 10.1117/1.JBO.31.7.077001 (PMC13371110; doi:10.1117/1.JBO.31.7.077001)
Supplement: Supplementary file 1 [file JBO_031_077001_SD001.pdf]

A sensor-integrated dual-clad fiber probe for OCT-guided retinal endolaser photocoagulation: Supplementary Material

This supplementary material provides information on the analysis, in addition to the results presented in the primary document.

1. ASSESSMENT OF A GRIN-LENSED FIBER PORBE APPROACH

In this section, we investigated the feasibility of using a GRIN lens fiber probe to meet the applicative requirements of the combined sensing system for retinal endolaser photocoagulation (REPC). Several commercially available GRIN lenses were evaluated through optical simulations in the commercial ray-tracing software ZEMAX OpticStudio. These simulations were used to determine whether the lenses could satisfy both the size constraints and the beam-shaping requirements of the target clinical application.

The GRIN rod lenses were selected from the product catalog and datasheet of GRINTECH GmbH (Jena, Germany) [1], with the primary selection criteria being design wavelength, lens diameter, and numerical aperture (NA). The selected GRIN lenses and their key specifications are summarized in Table S1. The same dual-clad fiber (DCF) (F-SMM900/007, Fibercore, Southampton, United Kingdom) used in this work was modeled at input. The dual-clad fiber (DCF) has a single-mode core with a mode-field diameter of ∼6 µm and a NA of 0.18, and a multimode first cladding with a diameter of 100 µm and a NA of 0.22. The input laser parameters in the simulations were defined based on the DCF core size and NA, and the propagation medium was defined with a refractive index of 1.33 (water).

Table S1. Summary of the commercially available GRIN lenses evaluated in optical simulations.

ID	Diameter (mm)	NA	Design Wavelength (nm)
GL-1	0.25	0.5	570
GL-2	0.35	0.2	670 - 1550
GL-3	0.35	0.5	670-1550
GL-4	0.5	0.2	670 - 1550
GL-5	0.5	0.5	670-1550

Since a DCF was used for the sensor design, the optical simulations were performed using a concentric beam configuration, in which both the surgical laser beam ($\lambda = 532$ nm) and the optical coherence tomography (OCT) beam (central wavelength $\lambda = 1060$ nm) share a common optical axis. This concentric configuration was selected to avoid the physical offset that would arise from attaching a separate fiber-based sensor to the side wall of an endolaser probe. Such an offset would not only increase the overall probe diameter, but would also introduce lateral and potentially angular displacements between the centers of the sensing and surgical laser beams, thereby increasing alignment complexity and potentially compromising the precision of beam delivery and sensing during the surgical procedure.

The simulated beam propagation for all evaluated lenses are presented in Fig. S1. The key simulation results for the different GRIN lenses are presented in Table S2. The primary parameters of interest were the back focal length of the OCT beam and the divergence (half-angle) of the surgical laser beam. The simulation results revealed two primary limitations. First, the NA of some GRIN lenses (e.g., GL-2 and GL-4, NA= 0.2) were too small to effectively guide the rapidly expanding surgical laser beam within the lens aperture. In these cases, substantial beam clipping

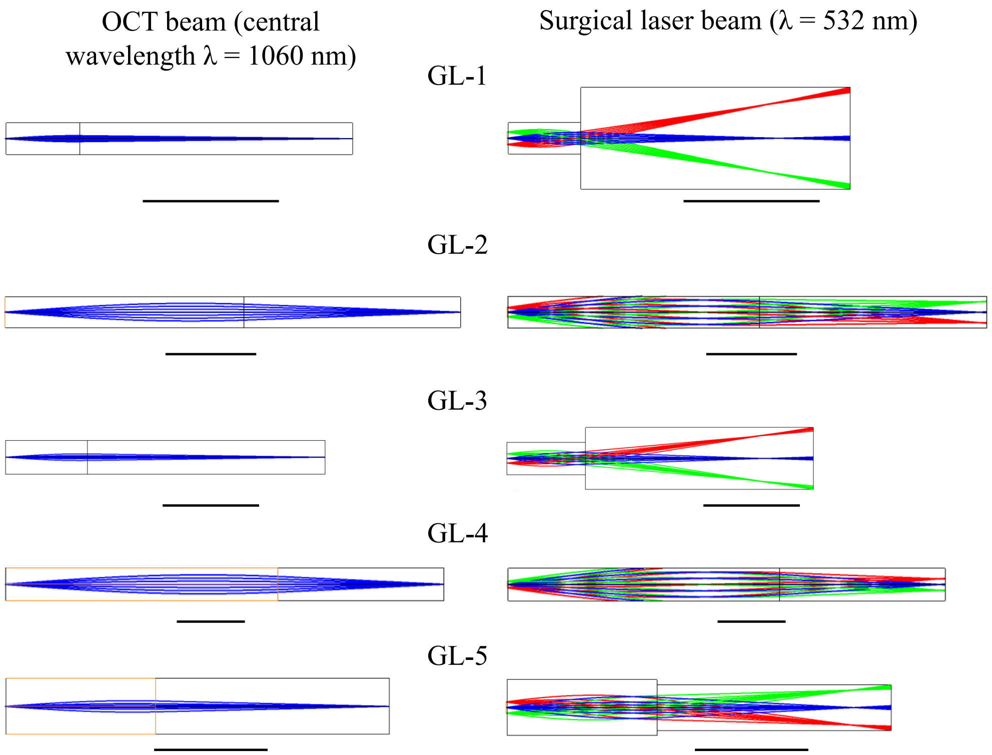

Fig. S1. ZEMAX simulation results of beam propagation through different GRIN lenses. Scale bar: 1 mm.

was observed, indicating a mismatch between the beam divergence from the DCF and the lens NA and diameter. Second, although lenses with larger NAs (e.g., GL-3 and GL-5) provided adequate beam-shaping performance, their diameters were excessively large, exceeding the inner diameter of a standard 23G needle (0.33 mm). This made it difficult to maintain the overall probe diameter within the 23G needle outer diameter (0.6 mm), even considering using customized tubing with a larger inner diameter.

Table S2. Summary of the optical simulation results. The dash ('-') in the 532 nm beam divergence (half-angle) column indicates that the beam size exceeded the lens aperture, making the calculation not feasible.

ID	Lens length (mm)	OCT back focal length (mm)	OCT image space NA	532 nm beam divergence (°)
GL-1	0.59	2.19	0.014	10.6
GL-2	2.75	2.49	0.049	-
GL-3	0.86	2.48	0.018	7.6
GL-4	4.20	2.57	0.063	-
GL-5	1.35	2.12	0.031	5.5

The simulation results indicate that, within the range of selected commercially available GRIN lenses and under the boundary conditions imposed by the target clinical application, a GRIN-lensed fiber probe could not readily satisfy both the size and beam shaping requirements. While a customized GRIN lens with reduced diameter and sufficiently large NA could in principle meet these requirements, the development of such a lens would introduce additional manufacturing complexity and cost for each design iteration. For these reasons, this approach was not pursued further, and a 3D nano-printed microlens approach was adopted in this work.

REFERENCE (also listed in the main text)

1. Grintech, “Grin rod lenses,” https://www.grintech.de/en/products/grin-rod-lenses/ (2025).
