# Supplementary material for: Sensor-integrated dual-clad fiber probe for OCT-guided retinal endolaser photocoagulation
Source: J Biomed Opt. 2026 Jul 15;31(7):077001. doi: 10.1117/1.JBO.31.7.077001 (PMC13371110; doi:10.1117/1.JBO.31.7.077001)
Supplement: Supplementary file 2 [file JBO_031_077001_SD002.pdf]

Supplementary Material 2: Alignment Tolerance and Coupling Efficiency Analysis of the Free-Space Fiber Coupling Setup

1. OVERVIEW

The free-space fiber coupling setup combines two optical paths into the dual-clad fiber (DCF) probe: the optical coherence tomography (OCT) path (single-mode fiber to DCF, 1060 nm) and the surgical laser path (multi-mode fiber to DCF, 532 nm). These two paths share the output collimator (Collimator 2) and the dichroic beamsplitter, but differ in their input fiber types, beam characteristics, and coupling mechanisms. The tolerance analysis was therefore conducted separately for each path, as illustrated in Fig.S1.

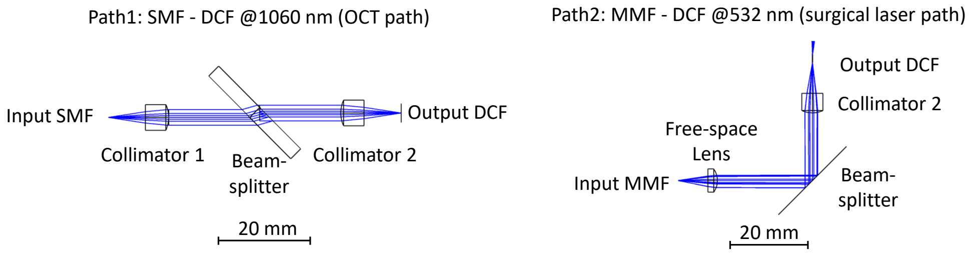

Fig. S1. ZEMAX simulation schematic of the two optical paths in the free-space fiber coupling setup. SMF: Single mode fiber. MMF: Multi-mode fiber. DCF: Dual-clad fiber.

2. OCT PATH TOLERANCE ANALYSIS

A. Simulation Method

Since the OCT path involves single-mode-to-single-mode core coupling, the tolerance analysis was performed in ZEMAX OpticStudio sequential mode using Physical Optics Propagation (POP), which accurately computes the overlap integral between the propagating field and the single-mode fiber acceptance mode. The coupling efficiency calculated by POP was transferred to the POPD merit function operand. The merit function (MF) was defined as:

$$MF = 1 - |POPD|$$

where a merit function value of zero corresponds to coupling efficiency of 1. Coordinate breaks with corresponding reverse solves were inserted into the optical design to introduce individual perturbations for each tolerance component.

B. Tolerance Components Analyzed

The following perturbation types were evaluated individually:

- Transverse decenter (X, Y) and axial shift (Z) of the input fiber (OCT sample arm fiber)
- Transverse decenter (X, Y), tilt (X, Y), and axial shift (Z) of the input fiber together with Collimator 1 as a rigid body (reflecting the physical configuration in which the input fiber is locked within the collimator housing and together being housed in a kinematic mount.)
- Transverse decenter (X, Y) and axial shift (Z) of the output fiber (DCF probe, reflecting plug-in and plug-out operations during probe exchange.)

The dichroic beamsplitter was not included as a perturbation source in this path, as it is located within a collimated beam section and produces negligible beam shift or angular deviation under typical mounting tolerances. The tolerance ranges for each component were defined based on the manufacturing specifications and bore eccentricity of the opto-mechanical cage mounts used in the setup.

C. Single-Parameter Sensitivity Results

The 1D sweep results (coupling efficiency as a function of each individual perturbation term) are shown in Fig.S2. The key findings are as follows:

- Transverse decenter of the output fiber was the most spatially sensitive term, as expected for single-mode-to-single-mode coupling into a fiber with a $\sim$6 µm mode-field diameter. Coupling efficiency dropped rapidly beyond ±2.5 µm decenter.
- Axial shift of the fiber tip relative to the collimator appeared sensitive in the unconstrained sweep. However, this represents a worst-case scenario that does not occur in practice, as the FC/APC connector constrains the physical axial shift to approximately ±10 µm, within which coupling efficiency remains high.
- Tilt of the input fiber together with Collimator 1 was the most angularly sensitive term, with coupling efficiency falling steeply beyond ±0.1°.

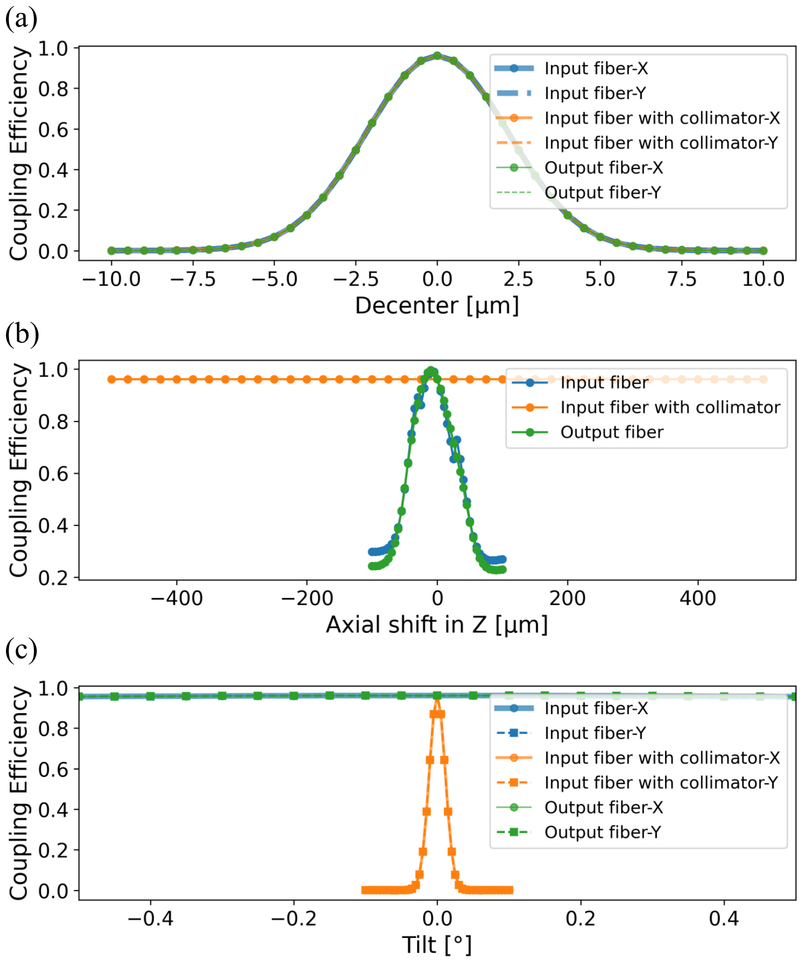

Fig. S2. OCT path single-parameter sensitivity analysis.

D. Monte Carlo Analysis

A Monte Carlo simulation of 2000 trials was run with all tolerance terms perturbed simultaneously within their defined ranges. The results are summarized below and shown in Fig.S3:

**Statistic**	**Coupling Efficiency**
Nominal	0.961
Mean	0.779
Standard deviation	0.140
5th percentile	0.499
95th percentile	0.956
Worst case	0.176

Table S1. Monte Carlo simulation results summary for the OCT path.

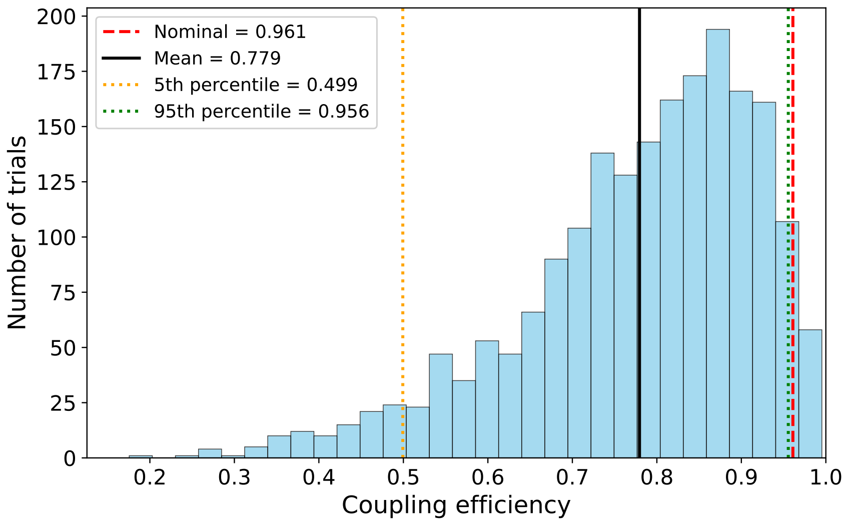

Fig. S3. Histogram of the Monte Carlo simulation results for the OCT path.

The measured single-direction coupling efficiency of the OCT path (77.86%, reported in Section 3.2 of the manuscript) is consistent with the simulated mean of 77.9%, validating the tolerance model. The wide spread of the distribution reflects the high sensitivity of single-mode coupling to transverse misalignment, and motivates our active alignment procedures during probe exchange.

3. SURGICAL LASER PATH TOLERANCE ANALYSIS

A. Simulation Method

The surgical laser path couples a multi-mode fiber (50 µm core, NA 0.22) into the multi-mode first cladding of the DCF (100 µm diameter, NA 0.22). Since multi-mode coupling is not governed by a single-mode overlap integral, the tolerance analysis was performed in ZEMAX OpticStudio non-sequential mode. Since the coated reflective surface of the beamsplitter faces the collimator in the actual setup, it was modeled for simplicity as a mirror surface angled at 45° in the optical path. To accurately represent the light entering DCF, the detector was positioned behind a section of optical fiber that has the same first cladding diameter and NA as the DCF, effectively acting as an acceptance filter. The coupling efficiency was defined as the ratio of the total optical flux collected by the detector to the source input power (1 W). The merit function operand NSDD was used to retrieve the total detector flux after initiating a non-sequential ray trace (NSTR).

B. Tolerance Components Analyzed

The following perturbation types were evaluated:

- Transverse decenter (X, Y) and axial shift (Z) of the input fiber tip
- Transverse decenter (X, Y) and axial shift (Z) of the free-space plano-convex lens
- Tilt (X, Y) of the dichroic beamsplitter
- Transverse decenter (X, Y) and axial shift (Z) of Collimator 2
- Transverse decenter (X, Y) and axial shift (Z) of the output fiber (DCF)

C. Single-Parameter Sensitivity Results

The 1D sweep results are shown in Fig.S4. The key findings are as follows:

- Tilt of the dichroic beamsplitter was identified as the most sensitive term. Even small angular deviations from the nominal 45° orientation caused a pronounced drop in coupling efficiency, as the beamsplitter tilt redirects the focused beam away from the DCF cladding acceptance cone.
- Transverse decenter of all other components produced relatively gradual efficiency reductions over the tested ranges (±50 µm), reflecting the more forgiving nature of multi-mode coupling into the 100 µm cladding.
- Axial shift similarly showed moderate sensitivity, with efficiency remaining above 0.85 for axial deviations within ±100 µm for most components.

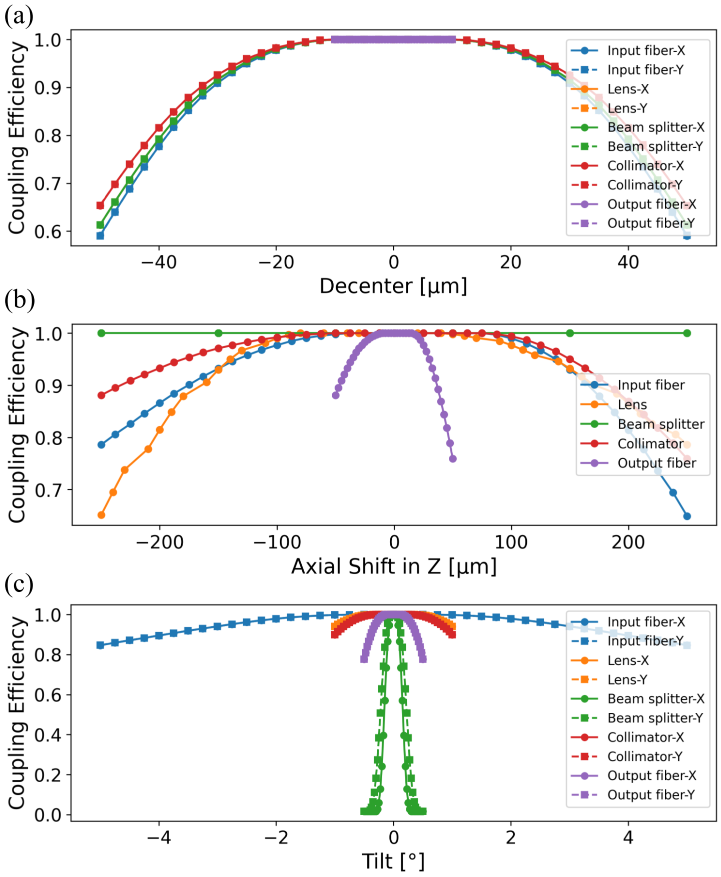

Fig. S4. Surgical laser path single-parameter sensitivity analysis.

D. Monte Carlo Analysis

A Monte Carlo simulation of 1000 trials was run with all tolerance terms perturbed simultaneously within their defined ranges. The results are summarized below and shown in Fig.S5:

Statistic	Coupling Efficiency
Nominal	0.990
Mean	0.863
Standard deviation	0.253
5th percentile	0.165
95th percentile	0.983
Worst case	0.000

Table S2. Monte Carlo simulation results summary for the surgical laser path.

The result shows that the large majority of trials produced coupling efficiency close to the nominal value. The small number of low-efficiency outliers were caused exclusively by large beamsplitter tilt perturbations. This confirms that beamsplitter angular stability is the primary robustness concern for the surgical laser path, and that the remaining components contribute negligible degradation under realistic mounting tolerances.

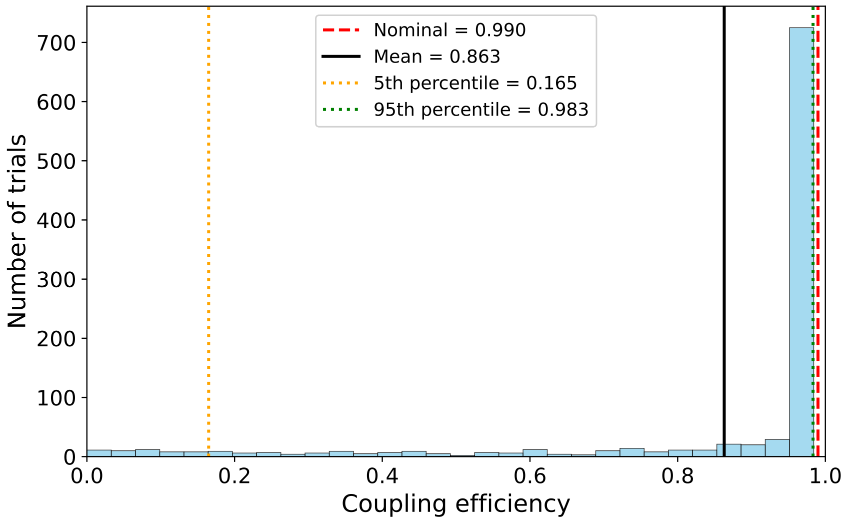

Fig. S5. Histogram of the Monte Carlo simulation results for the surgical laser path.

It should be noted that component-by-component optical power measurements revealed that the primary source of power loss in the surgical laser path is localized between Collimator 2 and the end facet of the DCF probe connector. This loss is most likely attributable to manual assembly tolerances during DCF probe fabrication, including residual angular or lateral misalignment at the fiber end facet and surface defects introduced during manual fiber tip polishing. This assembly-related loss is distinct from the alignment sensitivity of the free-space optical components characterized in the tolerance analysis above, and does not affect the conclusions of the sensitivity or Monte Carlo analyses, which concern the stability of the free-space coupling setup rather than the absolute loss of the system assembly. Furthermore, the long-term stability of the surgical laser path demonstrated in Section 3.2 (Fig. 6) in out manuscript confirms that this assembly-related loss is consistent and repeatable, and does not reflect a fundamental limitation of system robustness. Improving probe assembly precision in future iterations is expected to increase the surgical laser coupling efficiency toward the simulated nominal value.
