# Supplementary material for: Sensor-integrated dual-clad fiber probe for OCT-guided retinal endolaser photocoagulation
Source: J Biomed Opt. 2026 Jul 15;31(7):077001. doi: 10.1117/1.JBO.31.7.077001 (PMC13371110; doi:10.1117/1.JBO.31.7.077001)
Supplement: Supplementary file 3 [file JBO_031_077001_SD003.pdf]

Supplementary Material 3: ANSI Z136.1 Maximum Permissible Exposure Calculation for the iiOCT Probe

This supplementary document provides the detailed maximum permissible exposure (MPE) calculation for the optical coherence tomography (OCT) beam delivered by the instrument-integrated optical coherence tomography (iiOCT) probe, performed in accordance with ANSI Z136.1 (Safe Use of Lasers) standard [1]. The calculation supports the safety statement in Section 3.4 of the main manuscript, where the measured probe-tip power of 4.02 mW is compared against the derived MPE limit.

Since the iiOCT probe is operated intraocularly and the OCT beam bypasses the anterior optics of the eye (cornea and lens), we back-calculate the MPE from the retinal beam parameters to obtain the equivalent corneal power limit, noting that the ANSI corneal MPE is defined precisely to limit retinal irradiance and the comparison is therefore physically equivalent.

For the OCT wavelength (1060 nm) and exposure durations for iiOCT M-scan (1440 ms), the MPE radiant exposure is given by:

$$H_{\mathrm{MPE}} = 1.8 \times C_A \times C_E \times t^{0.75} \quad \mathrm{mJ/cm^2} \tag{S1}$$

At $\lambda = 1060$ nm, which falls in the 1050–1400 nm sub-range, the correction factor $C_A = 10^{0.002(\lambda-700)} = 5.01 \approx 5.0$. Here we used λ = 1050 nm as a conservative value.

The extended-source correction factor C_E accounts for the angular subtense of the retinal beam footprint referenced to the corneal plane via the ANSI reduced-eye model. The angular subtense α_s is:

$$\alpha_s = \frac{d}{f} = \frac{37.7\ \mu\mathrm{m}}{17000\ \mu\mathrm{m}} = 2.22\ \mathrm{mrad} \tag{S2}$$

where $d = 37.7$ μm is the OCT beam diameter at best focus on the retina, and $f = 17000$ μm is the ANSI reduced-eye posterior nodal distance.

The worst-case stationary exposure corresponds to one full iiOCT M-scan acquisition (1440 ms). We adopt $t = 2$ s as a conservative upper bound, which also covers any brief repositioning pause between acquisitions. During probe movement between imaging sites the beam sweeps continuously across the retina, so the local dwell time during probe movement is far shorter than 2 s. The stationary M-scan window therefore represents the true worst case. For $t = 2\ \mathrm{s} > 0.7$ s, ANSI specifies $\alpha_{\min} = 1.5$ mrad and $\alpha_{\max} = 100$ mrad. Since $\alpha_{\min} < \alpha_s < \alpha_{\max}$, the intermediate correction applies:

$$C_E = \frac{\alpha_s}{\alpha_{\min}} = \frac{2.22\ \mathrm{mrad}}{1.5\ \mathrm{mrad}} = 1.48 \tag{S3}$$

The MPE radiant exposure is then:

$$\begin{aligned} H_{\mathrm{MPE}} &= 1.8 \times C_A \times C_E \times t^{0.75} \\ &= 1.8 \times 5.0 \times 1.48 \times (2)^{0.75} \\ &= 1.8 \times 5.0 \times 1.48 \times 1.682 \\ &= 22.4\ \mathrm{mJ/cm^2} \end{aligned} \tag{S4}$$

The corresponding MPE irradiance is:

$$E_{\mathrm{MPE}} = \frac{H_{\mathrm{MPE}}}{t} = \frac{22.4\ \mathrm{mJ/cm^2}}{2\ \mathrm{s}} = 11.2\ \mathrm{mW/cm^2} \tag{S5}$$

For ocular exposure in the retinal hazard region, ANSI specifies a 7 mm limiting aperture representing the worst-case dilated pupil, giving an aperture area of:

$$A_{\text{ap}} = \pi \left(\frac{3.5 \text{ mm}}{10} \right)^2 = \pi \times (0.35 \text{ cm})^2 = 0.385 \text{ cm}^2 \tag{S6}$$

The maximum permissible power at the corneal-equivalent reference surface is therefore:

$$P_{\text{MPE}} = E_{\text{MPE}} \times A_{\text{ap}} = 11.2 \text{ mW/cm}^2 \times 0.385 \text{ cm}^2 = 4.31 \text{ mW} \tag{S7}$$

The measured probe tip power in the ex vivo experiment was $P = 4.02$ mW, therefore falls below the ANSI Z136.1 MPE of 4.31 mW.

We would also like to emphasize that the present work demonstrates the feasibility of the combined sensing concept and is not intended as a clinical-grade device. As part of future work we mentioned in Conclusion, improving the coupling efficiency of the free-space setup will allow the probe-tip power to be reduced further without compromising signal strength, which could further increase the safety margin. In addition, programmatic power attenuation during lateral probe movement between treatment sites could further limit retinal exposure to the brief acquisition windows required for iiOCT M-scan imaging. These considerations will be addressed in future development stages.

REFERENCE (also listed in main text)

1. Laser Institute of America, "American National Standard for Safe Use of Lasers, ANSI Z136.1," (2022). American National Standards Institute (ANSI).
